# The Practitioner’s Reference Book

**Published:** 1877-12

**Authors:** 


*The Practitioner's Reference Book.* Adapted to the use of the Physician, the Pharmacist, and the Student. By Richard J. Dunglison, M. D. Philadelphia: Lindsay & Blakiston. 1877. Buffalo: T. H. Butler. pp. 341. Price, $3.50.

